# GSK3β and mTORC1 Represent 2 Distinct Signaling Markers in Peripheral Blood Mononuclear Cells of Drug-Naive, First Episode of Psychosis Patients

**DOI:** 10.1093/schbul/sbac069

**Published:** 2022-06-27

**Authors:** Petros Petrikis, Alexandra Polyzou, Kyriaki Premeti, Argyro Roumelioti, Andreas Karampas, Georgios Georgiou, Dionysios Grigoriadis, George Leondaritis

**Affiliations:** Department of Psychiatry, Faculty of Medicine, School of Health Sciences, University of Ioannina, Ioannina, Greece; Department of Pharmacology, Faculty of Medicine, School of Health Sciences, University of Ioannina, Ioannina, Greece; Department of Pharmacology, Faculty of Medicine, School of Health Sciences, University of Ioannina, Ioannina, Greece; Department of Pharmacology, Faculty of Medicine, School of Health Sciences, University of Ioannina, Ioannina, Greece; Department of Psychiatry, Faculty of Medicine, School of Health Sciences, University of Ioannina, Ioannina, Greece; Department of Psychiatry, Faculty of Medicine, School of Health Sciences, University of Ioannina, Ioannina, Greece; European Molecular Biology Laboratory, European Bioinformatics Institute (EMBL-EBI), Wellcome Genome Campus, Cambridgeshire, UK; Department of Pharmacology, Faculty of Medicine, School of Health Sciences, University of Ioannina, Ioannina, Greece; Institute of Biosciences, University Research Center of Ioannina, Ioannina, Greece

**Keywords:** Akt, S6, mTOR, inflammation, insulin, anti- psychotics

## Abstract

**Background and Hypothesis:**

Schizophrenia is characterized by a complex interplay between genetic and environmental risk factors converging on prominent signaling pathways that orchestrate brain development. The Akt/GSK3β/mTORC1 pathway has long been recognized as a point of convergence and etiological mechanism, but despite evidence suggesting its hypofunction, it is still not clear if this is already established during the first episode of psychosis (FEP).

**Study Design:**

Here, we performed a systematic phosphorylation analysis of Akt, GSK3β, and S6, a mTORC1 downstream target, in fresh peripheral blood mononuclear cells from drug-naive FEP patients and control subjects.

**Study Results:**

Our results suggest 2 distinct signaling endophenotypes in FEP patients. GSK3β hypofunction exhibits a promiscuous association with psychopathology, and it is normalized after treatment, whereas mTORC1 hypofunction represents a stable state.

**Conclusions:**

Our study provides novel insight on the peripheral hypofunction of the Akt/GSK3β/mTORC1 pathway and highlights mTORC1 activity as a prominent integrator of altered peripheral immune and metabolic states in FEP patients.

## Introduction

Neuropsychiatric disorders, including schizophrenia, are characterized by a complex interplay between genetic and environmental risk factors. As such, dysregulation of prominent intracellular signaling pathways that integrate developmental, genetic, and environmental signals to orchestrate brain development and function has been highlighted as a key pathophysiological mechanism.^1–5^ One of the pathways that is increasingly recognized as highly relevant for neuropsychiatric disorders is the Akt/GSK3β/mTOR signaling pathway. This core pathway receives systemic input (ie, growth factors, cytokines, and neurotransmitters) as well as input from available nutrients, the energy status of cells, and stressful conditions to coordinate neuron growth and metabolism, survival, migration, dendrite and axon development, and neuronal connectivity.^4,5^

**Fig. 1.**
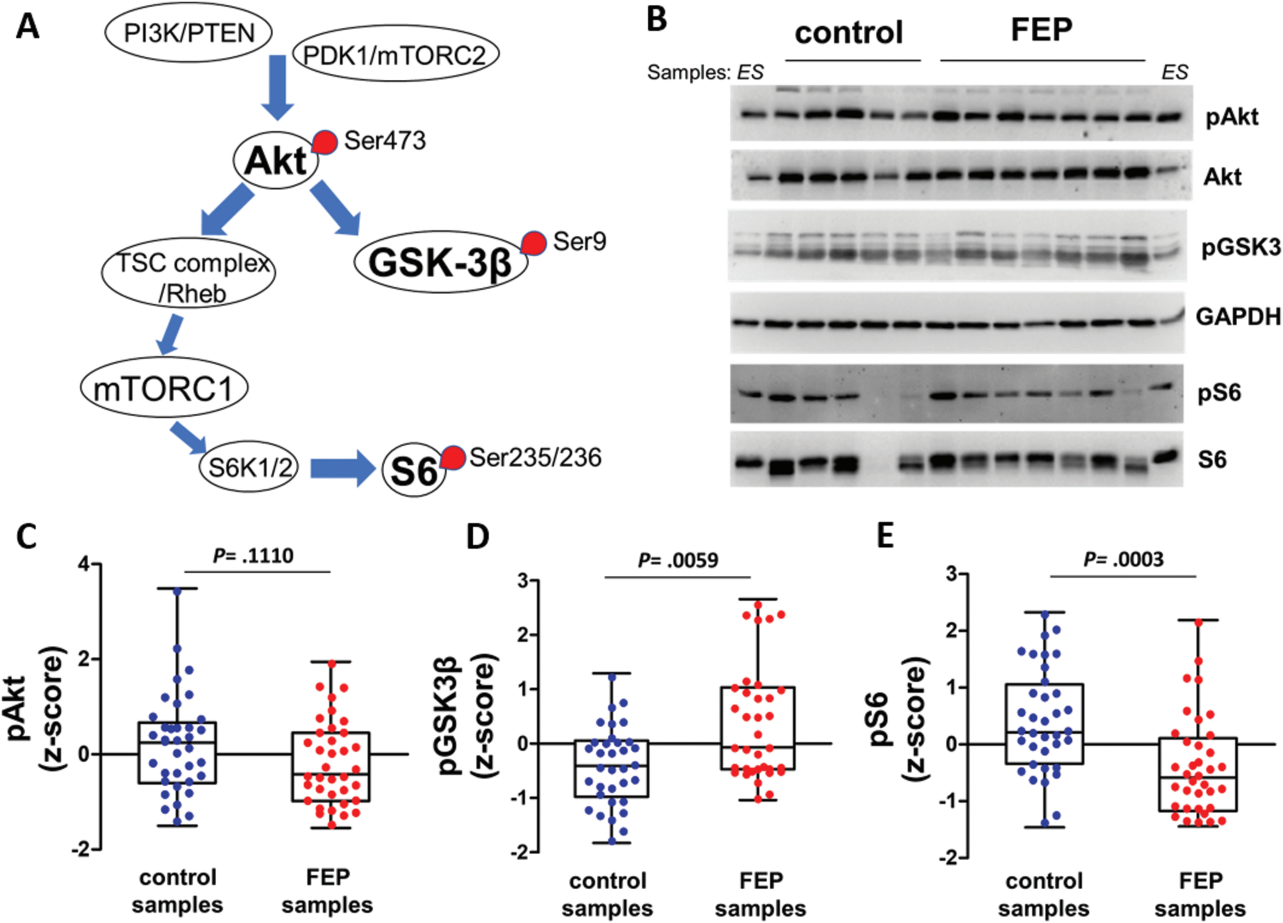
Activity of Akt/GSK3β/mTORC1 pathway in peripheral blood mononuclear cells (PBMCs) from first episode of psychosis (FEP) patients and controls. (A) PTEN and PI3K control the activation of Akt via phoshorylation by PDK1 and mTORC2. Akt, in turn, phosphorylates GSK3β, and, via TSC complex and Rheb, activates mTORC1 and S6K-phosphorylation of S6. Circles correspond to the phospho-epitopes measured. (B) Representative PBMC samples from controls (*n* = 5) and FEP patients (*n* = 7) were separated by sodium dodecyl-sulfate polyacrylamide gel electrophoresis and probed with the indicated antibodies. ES corresponds to external standard sample. (C–E) Comparisons of pAkt (C), pGSK3β (D) and pS6 (E) in FEP and control PBMC samples (*n* = 36). Statistical significances were analyzed by Mann-Whitney *U* tests.

Akt kinases represent prominent proteins in this signaling network. Upon engagement by receptors, they are phosphorylated and activated and subsequently phosphorylate GSK3β on Ser9 resulting in inhibition of GSK3β activity (figure 1A). In parallel, Akt indirectly activate mTORC1 kinase^6^ (figure 1A). Inhibition of GSK3β translates into positive regulation of GSK3β-dependent insulin and immune system responses, growth of axonal and dendritic branches, and synapse formation,^7^ whereas mTORC1 positively regulates anabolic reactions and neuron growth and differentiation, dendrite branching, and synaptic plasticity.^2^ mTORC1 via the ribosomal S6 kinase (S6K) controls phosphorylation of S6 ribosomal protein subunit (figure 1A), which, together with additional mTORC1 effectors, execute translational control over protein synthesis.^6^ Thus, phosphorylation levels of Akt, GSK3β, and/or S6 can be used to infer the functional signaling output of this pathway.^8^

The Akt/GSK3β/mTOR signaling pathway has a consistent multifaceted association with the pathophysiology of schizophrenia. Early seminal work showed that genetic variants of genes that result in decreased signaling output are associated with risk of schizophrenia.^9–11^ Furthermore, the Akt/GSK3β/mTOR pathway was found to represent a prominent therapeutic target of antipsychotic drugs (APDs) in the brain.^9,12,13^ More recently, independent studies have shown a hypofunction endophenotype (ie, reduced signaling output) in brain tissue of chronic schizophrenia patients.^14–17^ Of note, this hypofunction cannot be fully explained by the genetic background of schizophrenia,^18^ while confounding factors such as chronic illness, associated comorbidities, and long-term antipsychotic medication may influence these findings.^14,19^

Whether Akt/GSK3β/mTOR pathway hypofunction is an early event already established during the first episode of psychosis (FEP) can only be approached by assaying peripheral tissue. Schizophrenia can be viewed as a systemic disorder with parallel manifestations in both the brain and peripheral tissues.^20–22^ Although many studies have assessed expression of prominent genes of the Akt/GSK3β/mTORC1 pathway in peripheral blood mononuclear cells (PBMCs) from FEP patients,^23–26^ the coordinated signaling output of the pathway has not been described comprehensively so far.

In this report, we performed a systematic analysis of the phosphorylation status of Akt, GSK3β, and the downstream target of mTORC1, S6, in fresh PBMC samples of drug-naive FEP patients and control subjects. Our results suggest 2 distinct peripheral signaling endophenotypes during the first episode of psychosis. Hypofunction of mTORC1/S6K activity probably represents a stable state, whereas hypofunction of GSK3β is normalized upon clinical improvement after APD treatment.

## Materials and Methods

### Participants

Patients (*n* = 36) were recruited from the “Early Intervention in Psychosis Unit” of the Department of Psychiatry, University Hospital of Ioannina from May 2018 till April 2020. Inclusion criteria were as follows: (a) DSM-5 diagnosis of schizophrenia, schizophreniform disorder, or brief psychotic episode (follow-up after the completion of this study confirmed a diagnosis of schizophrenia for all patients); (b) patients were experiencing their first psychotic episode; (c) they were antipsychotic naive; and (d) they did not suffer from diabetes mellitus or had a known history of diabetes mellitus of any type. All patients had a complete physical examination and a urine test to exclude current substance use. Psychopathology was evaluated using the Positive and Negative Syndrome Scale (PANSS) and conducted by an experienced psychiatrist on the day of blood sample collection. The weight (kg) and height (m) of participants were measured, and their body mass index (BMI, kg/m^2^) was calculated. Patients’ psychopathology was reevaluated 6 weeks after the initiation of antipsychotic medication using the same scale and their weight and BMI were measured again. APDs administered in the study were olanzapine (*n* = 17) and risperidone (*n* = 19). The doses at the end of the 6-week period were 4–6 mg/day (risperidone) and 10–20 mg/day (olanzapine).

Healthy controls (*n* = 36) were recruited among Ioannina University students, hospital and university employees, and local enterprise employees. Mental health history was evaluated using the SCID NP (nonpatient) edition and only those without past or present history of mental disorder (psychotic, mood, and/or anxiety disorder) were included in the study. All control subjects were examined by an internist, were physically healthy, and were not taking any medications. Subjects with a history of diabetes mellitus, hypertension, cardiac disease, or thyroid disease were excluded from the study, as well as those who met the DSM-V criteria for alcohol or substance abuse. All control subjects were tested for the use of psychotropic substances with a urine test. All control subjects had BMI < 25.

The study was carried out in accordance with the Code of Ethics of the World Medical Association (Declaration of Helsinki). The study protocol was approved by the Ethics Committee of the University Hospital of Ioannina, University of Ioannina, Ioannina, Greece. All participants received detailed information about the aim of the study and gave informed consent.

### Western Blotting Analysis and Statistics

PBMCs were prepared within 4 hours from approximately 4 ml of blood collected into K_2_EDTA tubes (BD Biosciences). Whole blood was diluted with sterile PBS (Sigma-Aldrich), and the PBMCs were isolated by a density gradient centrifugation method using Ficoll Histopaque (supplementary methods).^27^

For immunoblotting, PBMC protein samples (10 μg), together with external standard (ES) samples (2 aliquots of 10-μg lysate from human A549 cells per gel), were mixed with 6× Laemmli loading buffer and processed as described previously.^8^ Primary and secondary antibodies and conditions for immunoblotting are given in supplementary methods.

For quantification, chemiluminescence signals from each gel were quantified by densitometry using FIJI software. Densitometry measurements for each sample were normalized to the average of 2 ES samples run on the same gel. Each normalized phospho-specific signal was expressed as a ratio against total expression of Akt (for pAkt), GAPDH (for pGSK3β), and S6 (for pS6). These were measured in sister gels or on the same gel and normalized accordingly to their corresponding ESs. The final normalized pAkt, pGSK3β, and pS6 values were imported into R (R version 4.1.0)^28^ using R studio (Version 1.4.1717),^29^ standardized as *z*-score,^30^ and then used for subsequent analyses. Statistical differences between control and FEP patient samples were assessed by nonparametric Mann-Whitney *U* tests. A multivariate exploratory analysis was conducted (supplementary tables 1 and 2; supplementary material) to assess the association between the status of the sample (control vs FEP) and the phosphorylation values. A similar workflow was followed to compare samples at baseline and after APD treatment from 32 patients because 4 patients were not available for blood samples. Spearman correlation testing was performed to determine the correlations between pAkt, pGSK3β, and pS6 and psychopathology after treatment. Wilcoxon matched pair tests were used for comparisons of pAkt, pGSK3β, and pS6 at baseline and after APD treatment. A *P*-value of less than .05 (2 tailed) was used for statistical significance.

Further details on methodology, design, assessment of reproducibility, and representative uncropped images of western blots can be found in supplementary methods and supplementary figure 1.

## Results

### Phosphorylations of Akt Downstream Effectors GSK3β and S6 Exhibit Reciprocal Changes in the PBMCs of Patients With First-Episode Psychosis

In total, we analyzed 36 FEP patient and 36 control blood samples. Inclusion criteria were established according to previous studies.^22,31^ There were no significant differences in sociodemographic data (age, smoking, and BMI) between control and patient groups (table 1).

**Table 1.** Sociodemographic of the Study Sample and Psychopathology and Clinical Characteristics of Control and Drug-Naive First Episode of Psychosis Patients at Baseline and After Antipsychotic Drug Treatment

	Control Subjects (*n* = 36)	Patients (Baseline) (*n* = 36)	Patients (APD Treatment)	Statistics
Gender (male/female)	24/12	24/12	n.a.	^a^ *χ* ^2^ = 0; *P* = 1
Smokers/nonsmokers	18/18	18/18	n.a.	^a^χ ^2^ = 0; *P* = 1
Age (y)	31.4 (8.9)	31.3 (8.1)	n.a.	^a^ *U* = 646.0; *P* = .9865
BMI (kg/m^2^)	22.9 (1.5)	22.6 (1.1)	23.3 (1.3)	^a^ *t* = 0.79; *P* = .4292 ^b^***t*=5.8**; ***P*<.0001**
Diagnosis				
Schizophrenia		4		
Schizophreniform disorder		27		
Brief psychotic episode		5		
Severity of symptoms (PANSS scores)^c^		Baseline	APD treatment	Statistics
**PANSS-P**		31.4 (2.04)	7.6 (1.1)	^b^ ***t*** **=** **63.8**; ***P*<.0001**
**PANSS-N**		20.6 (3.2)	18.3 (2.9)	^b^ ***t*** **=** **3.6**; ***P*=.0009**
**PANSS-G**		25.1 (2.8)	19.2 (2.4)	^b^ ***t*** **=** **9.8**; ***P*<.0001**
**PANSS-T**		77.0 (5.7)	45.1 (5.1)	^b^ ***t*** **=** **27.9**; ***P*<.0001**
**DUP (wk)**		14.6 (7.6)	n.a.	n.a.
APD (RIS/OLA)^c^		n.a.	19/17	n.a.

*Note*: APD, antipsychotic drugs; BMI, body mass index; DUP, duration of untreated psychosis; n.a., not applicable; PANSS, Positive and Negative Syndrome Scale (PANSS-P, positive; PANSS-N, negative; PANSS-G, general psychopathology; PANSS-T, total score). Values are means with the SD in parentheses. Bold indicates statistical significance.

^a^Statistics between control and patients (unpaired *t*-test or Mann-Whitney *U* test).

^b^Statistics between patients baseline and APD treatment (paired *t*-test).

^c^APD, antipsychotic drug; RIS, risperidone; OLA, olanzapine. Chloropromazine equivalents were in the range of 200–400 mg.

We analyzed phosphorylations of Akt, GSK3β, and S6 in PBMC protein extracts using phospho-specific antibodies against Ser473-Akt, Ser9-GSK3β, and Ser235/236-S6^8^ (figure 1A). We did not detect statistical difference between normalized and *z*-score transformed pAkt in FEP patients compared with controls (Mann-Whitney *U* test, *P* = .0872) (figure 1B and 1C). In contrast, we detected significant increases in pGSK3 values from FEP patients compared with controls (36% increase, Mann-Whitney *U* test, *P* = .0059) (figure 1B and 1D). We next assessed the pS6 values and detected a significant decrease in FEP patients (42% reduction, Mann-Whitney *U* test, *P* < 0.0001), indicating attenuation of mTORC1 and S6K activity (figure 1B and 1E). These reciprocal changes of pGSK3β and pS6 in FEP patients’ PBMCs suggest that Akt signaling is dysregulated peripherally at the time of the first psychotic episode in drug-naive patients.

### Correlations of PBMC Akt, GSK3β, and S6 Phosphorylations and Psychopathology of FEP Patients

**Fig. 2.**
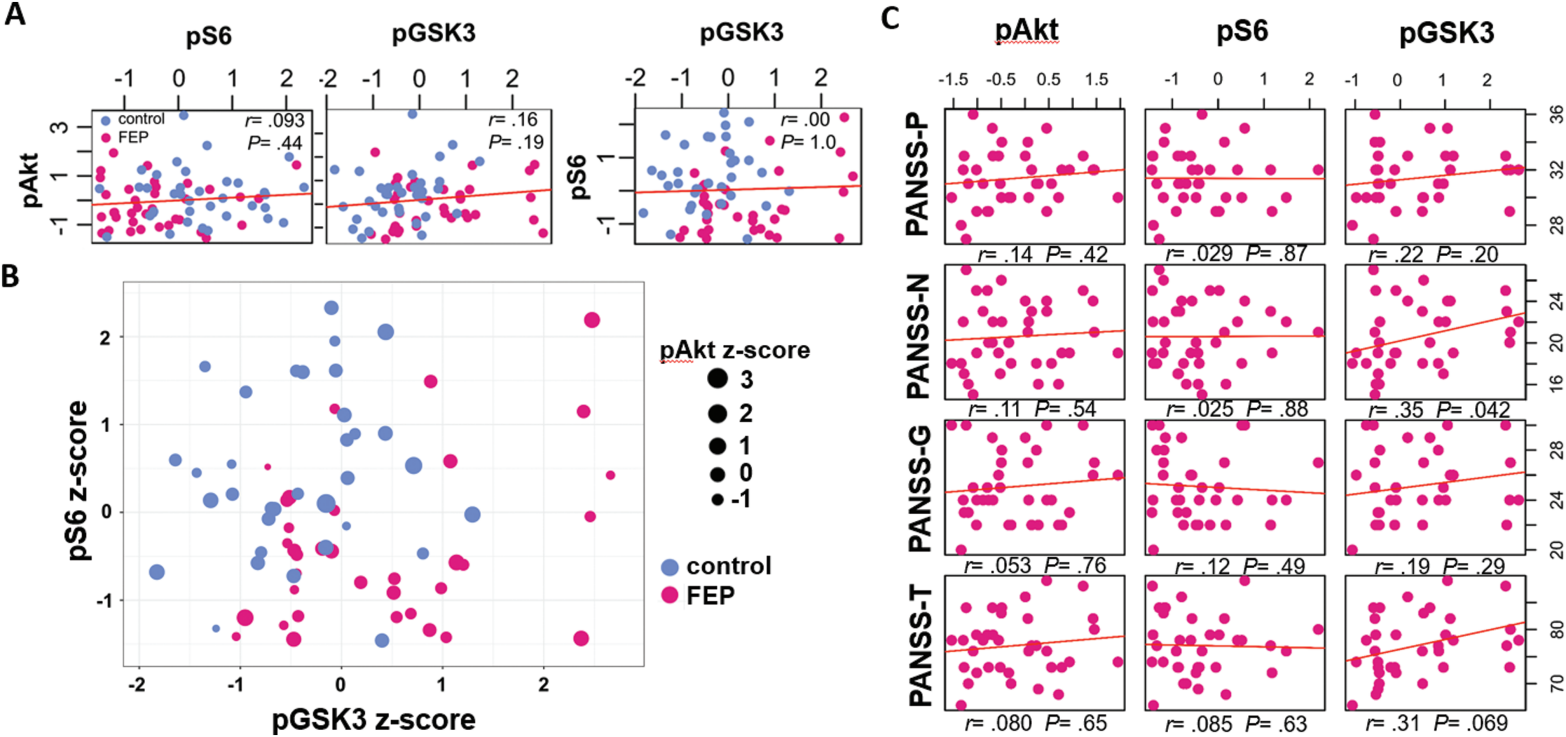
Correlations of peripheral blood mononuclear cell (PBMC) Akt/GSK3β/mTORC1 pathway activity with psychopathology. (A) Correlations between pAkt, pS6, and pGSK3β in pooled samples. Control and first episode of psychosis (FEP) samples are indicated. Spearman correlation coefficients (*r*) and *P* values are indicated. (B) Scatterplot of pS6 vs pGSK3β vs pAkt from both groups. pAkt values are represented by the indicated dot size gradient. (C) Correlations of pAkt, pS6, and pGSK3β with positive (-P), negative (-N), general (-G), and total (-T) Positive and Negative Syndrome Scale (PANSS) scores in the group of FEP patients. Spearman correlation coefficients (*r*) and *P* values are indicated.

Considering the dynamics of signaling in the Akt/GSK3β/mTORC1 pathway, it is reasonable to expect a certain level of correlation between pAkt, pGSK3β, and pS6 (figure 1A). Thus, we first tested correlations between pAkt, pGSK3β, and pS6 in all samples from both control and FEP patient groups using Spearman correlation analyses. Although we did not detect any statistically significant correlations in pooled samples (figure 2A), a trend for weak correlation between pAkt and pGSK3β was observed in control (*r* = .31, *P* = .079), but not FEP samples (*r* = .23, *P* = .19). Strong positive correlations of pAkt, pGSK3β, and pS6 are evident upon challenge of PBMCs with growth factors or pharmacological compounds in vitro^32^; our results suggest that this is not the case for fresh unchallenged PBMCs under basal conditions in vivo.

Visualization of all pAkt, pGSK3β, and pS6 values on a single graph (with pAkt values represented by dot size) revealed a distinct high pGSK3β/low pS6 pattern in FEP patients, compared with low pGSK3β/high pS6 controls, while FEP patients also had slightly lower pAkt values (figure 2B). To explore whether all 3 phosphorylation values and/or their interactions significantly influence control vs FEP state, we conducted a regression logistic analysis (supplementary methods and supplementary tables 1 and 2). The regression analysis revealed that all 3 values were significant including the small reduction (15%) of pAkt in FEP patients (figure 1A and supplementary table 1); adding to the analysis, all their interactions did not further improve significance (supplementary table 2). Although this approach is weakened due to the small sample size, it validated the distinct high pGSK3β/low pS6 pattern in the background of somewhat lower pAkt values in FEP patients (figure 2B). Evidently, further studies with larger sample sizes including additional pertaining factors will be needed to verify this issue.

Since PBMCs of FEP patients are characterized by distinct changes in pGSK3β and pS6, we further tested the correlation of all phosphorylation values with the PANSS scores (table 1). pAkt and pS6 did not correlate with PANSS-P, PANSS-N, PANSS-G, or PANSS-T scores (figure 2C). Surprisingly however, pGSK3β values showed a trend for positive correlations with the PANSS-N (*r* = .35, *P* = .042) and PANSS-T (*r* = .31, *P* = .069) score. Thus, of all signaling markers in the Akt pathway, only pGSK3β shows a marginal positive correlation with the severity of symptoms of psychopathology.

### PBMCs GSK3β Phosphorylation, but not S6 Phosphorylation, Is Normalized After Antipsychotic Treatment in FEP Patients

APDs are the mainstay of treatment in schizophrenia patients, while the Akt/GSK3/mTORC1 pathway has been implicated in APD mechanisms of action and therapeutic effects.^9,12,13^ Thus, we proceeded in analysis of PBMC samples of 32 patients that were available after treatment with olanzapine or risperidone for 6 weeks. Comparison between the PANSS scores at baseline and after treatment showed that all patients were clinically improved (table 1).

**Fig. 3.**
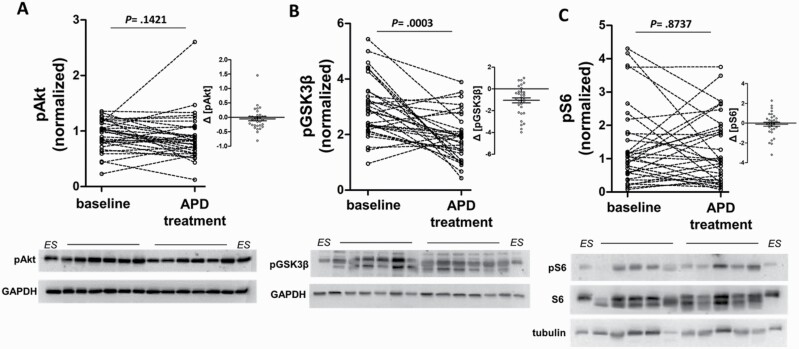
Effect of antipsychotic drug (APD) treatment on peripheral blood mononuclear cell (PBMC) Akt/GSK3β/mTORC1 pathway activity in first episode of psychosis (FEP) patients. (A–C) Paired comparisons of pAkt (A), pGSK3β (B), and pS6 (C) in PBMCs from FEP patients (*n* = 32) at baseline and after APD treatment. Statistical significances were assessed by Wilcoxon matched pairs tests. Right insets show the distribution of pAkt, pGSK3β, and pS6 changes after treatment (Δ[pAkt], Δ[pGSK3β], and Δ[pS6], respectively), and bottom western blot images show representative samples from patients (*n* = 5 or 6) before and after treatment, together with external standard samples (ES), probed with the indicated antibodies.

We detected a small reduction of pAkt in FEP patients after APD treatment (6% reduction), but no statistical difference between the 2 groups (Wilcoxon matched pairs test, *P* = .1421) (figure 3A). By contrast, we detected a significant decrease of pGSK3β values in FEP patients after APD treatment compared with baseline (36% decrease, Wilcoxon matched pairs test, *P* = .0003) (figure 3B). Overall, 78% of patients exhibited decreased phosphorylation of GSK3β after APD treatment, whereas the rest responded with increases of pGSK3β (figure 3B, inset). When we assessed the pS6 values we found no significant differences after APD treatment (Wilcoxon matched pairs test, *P* = .8737) (figure 3C).

Given the trend for correlation of pGSK3 with clinical psychopathology at baseline (figure 2C), we sought to further characterize this relationship after APD treatment. To control for the reduced number of patients included in the baseline vs APD treatment comparison, we reiterated the data set for the 32 patients at baseline and we found additional marginal associations of pGSK3β with PANSS-G and PANSS-T scores (supplementary table 3). However, in multiple analyses, there was no significant correlation of pGSK3β (nor pAkt or pS6) with PANSS scores after APD treatment (supplementary table 4). Furthermore, changes in pGSK3 (Δ[pGSK3]) did not show any correlation with the change in PANSS scores (Δ[PANSS]) of patients after APD treatment (supplementary table 5). We conclude that, although the decrease of pGSK3β after APD treatment, corroborates key findings of the comparison between drug-naive FEP patients and controls (figures 1B and 2C), changes in pGSK3, per se, do not associate with the overall extent of improvement of clinical psychopathology after APD treatment in our patient group.

## Discussion

Current research suggests that some of the underlying biological processes of schizophrenia have already been established even before the manifestation of the first psychotic episode.^33,34^ This is reminiscent of the imbalance of antioxidant and inflammatory responses as well as impaired glucose homeostasis detected peripherally in FEP patients.^35,36^ Primary peripheral cells represent the most relevant cellular model to assess the status of signaling pathways in FEP patients in real time.^20^ PBMCs are easily accessible and, additionally, bear the imprint of metabolic and immune system alterations detected in patients.^20,37^ Here, by comprehensively analyzing the level of phosphorylations that constitute the signaling output of the Akt/GSK3β/mTORC1 pathway in fresh PBMCs from FEP patients, we provide new insight to the previously proposed hypofunction of the pathway in schizophrenia.

We did not detect statistically significance difference of total Akt S473 phosphorylation in PBMCs from FEP patients compared with controls (figure 1). Nevertheless, the small reduction of 15% in total pAkt levels in PBMCs from FΕP patients might be relevant as suggested by our logistic regression analyses (figure 2B and supplementary table S1). Of note, previous work assessing Akt1 expression in PBMCs has given contradictory results, with some studies suggesting increase,^23^ decrease,^23,25^ or no changes^26^ in patients. It should be stressed out that we measured the phosphorylation of all Akt isoforms (Akt1, 2, and 3), which are all expressed in human PBMCs.^23^ Besides Akt1 variants,^9,10^ recent GWAS studies have also suggested Akt3 as a susceptibility locus for schizophrenia.^38^ When we studied the effects of APD treatment, the mean change in Akt phosphorylation was negligible (figure 3). Conclusively, our results suggest that, at least in PBMCs, there is no gross hypofunction at the level of pan-Akt isoform phosphorylation in drug-naive FEP patients.

GSK3β is one of the main substrates of Akt that has been studied extensively in psychiatric diseases. Surprisingly, when we studied pGSK3β in PBMCs from drug-naive FEP patients we found a significant 36% increase compared to control subjects (figure 1). Changes in pGSK3β are likely to be cell type or time dependent. For example, decreases of total and/or phospho-Ser9-GSK3β were reported in PBMCs, lymphoblasts, and brain tissue from chronic schizophrenia patients,^9,39,40^ in some but not all studies.^41,42^ Decrease of pGSK3β has also been found in platelets from APD-free patients.^43^ On the other hand, large increases (3-fold) of pGSK3β have been reported in fresh PBMCs from Li-free bipolar patients compared with control subjects.^44^ Thus, increased PBMC pGSK3β seems to represent a shared feature of drug-naive FEP and Li-free bipolar disorder patients.

The increased pGSK3β in PBMCs from FEP patients might reflect disease progression and/or tissue-specific differences. It is well established that GSK3β in its active form (ie, low p-Ser9-GSK3β) acts as a crucial positive regulator of proinflammatory cytokines including IL1β, IL6, TNF-α, and INF-γ.^7^ The relative inhibition of PBMC GSK3β (ie, high p-Ser9-GSK3β) that we detected in the FEP could mirror an innate immune cell compensatory response to the well-described peripheral proinflammatory state in FEP patients, which includes upregulation of IL1β and IL6.^36,45^ GSK3β has a pleiotropic role in balancing inflammation in both the periphery and the brain, and negative modulation of GSK3 activity appears to be a crucial step during the regulatory events governing the resolution of inflammation.^46^

Importantly, PBMC pGSK3β probably represents a peripheral signaling endophenotype that associates with psychopathology. First, pGSK3β levels in drug-naive FEP patients exhibited a promiscuous association with the severity of psychopathology (figure 2). We believe that analysis of larger groups of patients including additional factors will reveal the true extent of association. Second, risperidone or olanzapine treatment consistently normalized pGSK3β levels in most patients (figure 3). This might be, at least partially, due to a direct effect as APDs, including risperidone, have been shown to reduce Akt downstream signaling in PBMCs in vitro.^47^ Although we failed to detect association of the pGSK3β decrease after APD treatment to the clinical improvement (ie, decrease of PANSS scores), it is likely that such an association will be revealed by analyzing larger samples.

Our analyses of mTORC1/S6K signaling and specifically phosphorylation of S6 in PBMCs, revealed reduced pSer235/236 S6 levels in FEP patients compared with controls (figure 1). This is the first direct demonstration, according to our knowledge, that mTORC1/S6K signaling is attenuated in peripheral tissue samples from FEP patients. Since pS6 levels do not change after APD treatment (figure 3), the downregulation of PBMC mTORC1/S6K activity in FEP patients probably represents a signaling endophenotype which is stable, unlike the case of pGSK3β, and perhaps is associated with the pathophysiology of schizophrenia. Accordingly, recent independent studies support the notion of mTOR hypofunction in schizophrenia patients. For example, lower levels of mTOR and the GβL subunit of both mTORC1 and mTORC2 complexes, reduced p-Ser2448 mTOR, and reduced pSer235/236 S6 have all been readily detected in postmortem studies.^15–17^

mTOR operates in 2 distinct complexes and is recognized as a crucial protein for human cancer, metabolic diseases, and neurodevelopmental disorders.^2,5,6^ Furthermore, mTOR is a core signaling hub that bears connections to both the immune and metabolic responses.^6,48^ Our results suggest that PBMC mTORC1 activity may be the integrator of altered peripheral immune and metabolic states in FEP patients. It is important that, although peripheral metabolic and immune abnormalities might be a feature of many neuropsychiatric conditions, the functional links between the 2 features is likely to be more prominent in schizophrenia.^37,49^ Highlighting our findings on the hypofunction of mTORC1/S6K/S6 in PBMCs from FEP patients, p70S6K, the kinase that phosphorylates S6 ribosomal subunit, is the only gene altered in PBMCs from treatment-resistant chronic schizophrenia patients.^19^ These results combined suggest that the activity and regulation of mTORC1/S6K/S6, as well as other downstream effectors of mTOR complexes, are promising signaling endophenotypes to be further dissected in longitudinal studies in FEP patients.

There were some limitations to our study. First, our sample size is quite small, and compliance of medication was only indirectly assessed by clinical improvement. Further studies with larger samples are needed to clarify the exact association as well as the relationship of pGSK3β changes after specific APD treatment to the psychopathology and clinical improvement of patients. Second, another limitation is that we analyzed fresh PBMC samples to capture an “all-inclusive” snapshot of the activity of the pathway in vivo at a single time point after treatment. An alternative approach would have been to analyze cultured patient PBMCs.^32^ This is important, particularly for highlighting functional cellular endophenotypes focusing on new drug discovery,^20^ but it is likely that changes in PBMC signaling kinases might have been missed upon culture, as has been shown for pGSK3β.^44^

In summary, our results provide novel insight on the peripheral hypofunction of the Akt/GSK3β/mTORC1 pathway, which has long been proposed as a point of convergence and etiological mechanism for schizophrenia. We have revealed 2 distinct peripheral signaling endophenotypes in FEP patients that warrant further research aiming at discovering novel markers for disease progression and/or the therapeutic response to pharmacotherapy.

## Supplementary Material

sbac069_suppl_Supplementary_Materials
