# Supplementary material for: GSK3β and mTORC1 Represent 2 Distinct Signaling Markers in Peripheral Blood Mononuclear Cells of Drug-Naive, First Episode of Psychosis Patients
Source: Schizophr Bull. 2022 Jun 27;48(5):1136–44. doi: 10.1093/schbul/sbac069 (PMC9434466; doi:10.1093/schbul/sbac069)
Supplement: sbac069_suppl_Supplementary_Materials [file sbac069_suppl_supplementary_materials.pdf]

GSK3β and mTORC1 represent two distinct signalling markers in peripheral blood mononuclear cells of first-episode of psychosis patients

Petros Petrikis [1]*, Alexandra Polyzou [2], Kyriaki Premeti [2], Argyro Roumelioti [2], Andreas Karampas [1], Georgios Georgiou [1], Dionysios Grigoriadis [3] and George Leondaritis [2,4]*

[1] Department of Psychiatry, Faculty of Medicine, School of Health Sciences, University of Ioannina (UOI), P.O. Box 1186, 45110 Ioannina, Greece

[2] Department of Pharmacology, Faculty of Medicine, School of Health Sciences, University of Ioannina, 45110 Ioannina, Greece

[3] European Molecular Biology Laboratory, European Bioinformatics Institute (EMBL-EBI), Wellcome Genome Campus, Hinxton, Cambridgeshire, CB10 1SD, UK.

[4] Institute of Biosciences, University Research Center of Ioannina, 45110 Ioannina, Greece

* to whom correspondence should be addressed: gleondar@uoi.gr or ppetrikis@hotmail.gr

Supplementary Material

Including Supplementary Tables S1-S5, Supplementary Figure 1 and Supplementary Methods

Supplementary Tables

Supplementary Table 1. Logistic regression analysis results. This logistic analysis was calculated to assess the effects of pAkt, pGSK3 and pS6 phosphorylation values as continuous variables to the independent variable of the samples' state (control vs FEP).

	Wald statistics				
	β [a]	SEβ	z	p	Significance
Intercept	-0.1938	0.3214	-0.6030	0.5464	-
**Predictors**					
**pS6**	1.2154	0.3744	3.2470	0.0012	**
**pGSK3**	-1.5442	0.4593	-3.3620	0.0008	***
**pAkt**	0.7302	0.3522	2.0740	0.0381	*
AIC: 70.46					

[a] β: beta regression coefficient, SEβ: Standard Error of β, z: Wald z value, p: Wald p-value.

[b]AIC: Akaike information criterion

Supplementary Table 2. Logistic regression analysis results. This logistic analysis was calculated to assess the effects of pAkt, pGSK3 and pS6 phosphorylation values and their interactions as continuous variables to the independent variable of the samples' state (control vs FEP).

	Wald Statistics				
	β [a]	SEβ	z	p	Significance
Intercept	-0.0202	0.3979	-0.0510	0.9595	-
**Predictors**					
**pS6**	1.5479	0.4824	3.2090	0.0013	**
**pGSK3**	-1.8131	0.5792	-3.1300	0.0018	**
**pAkt**	1.1950	0.4646	2.5720	0.0101	*
**pS6:pGSK3**	-0.8681	0.6198	-1.4010	0.1613	ns
**pS6:pAkt**	0.7425	0.5816	1.2770	0.2018	ns
**pGSK3:pAkt**	0.1039	0.5133	0.2020	0.8396	ns
**pS6:pGSK3:pAkt**	-0.3282	0.5011	-0.6550	0.5124	ns
[b] AIC: 72.19					

[a] β: beta regression coefficient, SEβ: Standard Error of β, z: Wald z value, p: Wald p-value.

[b]AIC: Akaike information criterion

Supplementary table 3. Correlation of pGSK3β with severity of psychopathology (PANSS scores) at baseline. Spearman's correlation coefficients (*r*) and *P* values (in parentheses) are indicated. Note that this corresponds to reanalysis of the subgroup of 32 patients from Figure 2C that were available for follow-up and it highlights weak correlations of pGSK3β baseline levels with PANSS-G and PANSS-T scores (in bold). No significant differences were found for pAkt and pS6 correlations compared to data in Figure 2C.

	**severity of psychopathology**			
	**PANSS-P**	**PANSS-N**	**PANSS-G**	**PANSS-T**
**Phospho-protein**				
**pGSK3β**	*r*=.244 (*P*=.185)	*r*=-.265 (*P*=.149)	***r*=-.370 (*P*=.040)**	***r*=-.367 (*P*=.042)**

Supplementary table 4. Correlation of pAkt, pGSK3β and pS6 with severity of psychopathology (PANSS scores) after APD treatment (n=32). Spearman's correlation coefficients (*r*) and *P* values (in parentheses) are indicated.

	**severity of psychopathology**			
	**PANSS-P**	**PANSS-N**	**PANSS-G**	**PANSS-T**
**Phospho-protein**				
**pAkt**	*r*=.045 (*P*=.8062)	*r*=.151 (*P*=.4089)	*r*=.082 (*P*=.6549)	*r*=.185 (*P*=.3102)
**pGSK3β**	*r*=.115 (*P*=.5323)	*r*=-.063 (*P*=.7306)	*r*=-.126 (*P*=.4916)	*r*=-.074 (*P*=.6851)
**pS6**	*r*=-.056 (*P*=.7609)	*r*=.159 (*P*=.3860)	*r*=.132 (*P*=.4715)	*r*=.141 (*P*=.4408)

Supplementary table 5. Correlation of changes in pAkt, pGSK3β and pS6 (Δ[pAkt], Δ[pGSK3β], Δ[pS6], respectively) with changes in severity of psychopathology (Δ[PANSS] scores) after APD treatment (n=32). Spearman's correlation coefficients (*r*) and *P* values (in parentheses) are indicated.

	**Change in severity of psychopathology**			
	**Δ[PANSS-P]**	**Δ[PANSS-N]**	**Δ[PANSS-G]**	**Δ[PANSS-T]**
**Change in phospho-protein**				
**Δ[pAkt]**	*r*=.030 (*P*=.8684)	*r*=.108 (*P*=.5544)	*r*=-.198 (*P*=.2768)	*r*=-.089 (*P*=.6268)
**Δ[pGSK3β]**	*r*=.210 (*P*=.2481)	*r*=.050 *P*= (.7846)	*r*=.013 (*P*=.9441)	*r*=.111 (*P*=.5446)
**Δ[pS6]**	*r*=.332 (*P*=.0627)	*r*=-.151 (*P*=.4088)	*r*=.089 (*P*=.6253)	*r*=.131 (*P*=.4474)

Supplementary figure 1

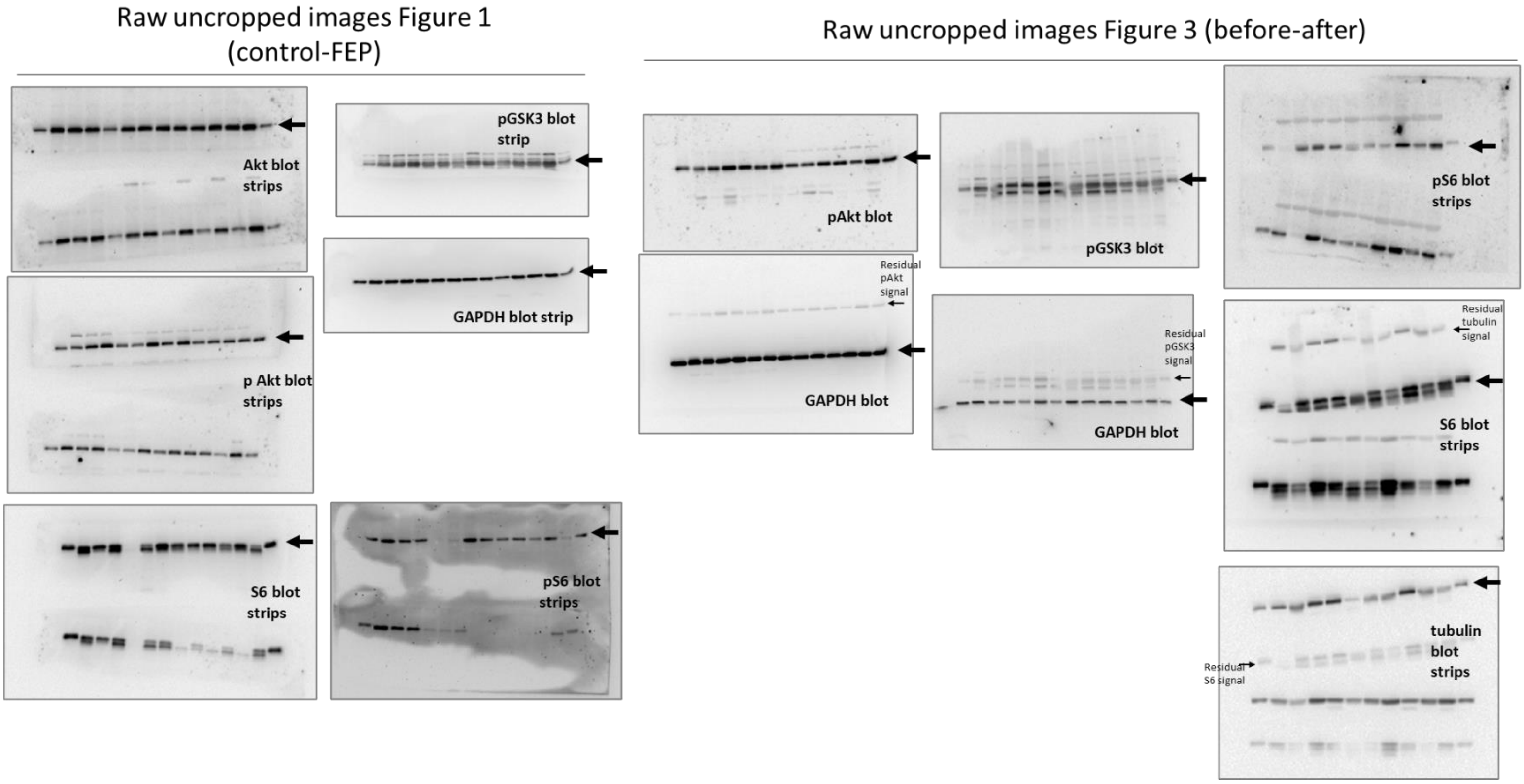

Supplementary figure 1. Uncropped WB images related to figure 1B and figure 3A-C. Large arrows denote the group of samples used in the main manuscript figures. Small arrows denote residual signal from previous blotting with different antibodies. Note that in some cases (e.g., S6/pS6, control-FEP), lower parts of two gels with different groups of samples were blotted on the same membrane. In other cases (e.g., Akt/p-Akt, control-FEP and other images), membrane strips with different groups of samples were imaged simultaneously. In the before-after group, whole membranes or cut strips were first probed for a signaling marker and subsequently for GAPDH or tubulin. For technical details on design and methodology please refer to Supplementary Methods.

Supplementary Methods

PBMCs isolation and protein extraction

Briefly, diluted whole blood was gently layered on the top of Ficoll Histopaque (4ml) in a 15ml centrifuge tube and was centrifuged at 1800rpm for 10min (20°C). The cell layer containing PBMCs at the interface between plasma and Ficoll was transferred to a clean tube and was washed with sterile PBS, with sequential centrifugation steps at 1600 rpm and then at 1400 rpm for 10 min each (20°C). The washed PBMC pellet was snap-frozen in liquid nitrogen and stored at -80°C. Stored PBMC pellets were thawed on ice and lysed with lysis buffer (20mM Bicine, pH=7,6, 0,6% w/v CHAPS) supplemented with phosphatase inhibitors (2mM sodium fluoride, 2mM sodium orthovanadate, 2mM beta-glycero phosphate, 2mM sodium molybdate), 0.2mM PMSF and 0,4% Protease Inhibitor cocktail 3 (539134, Calbiochem). After solubilization of proteins and centrifugation at 15000 rpm for 20min (4°C) to remove particulate material, the cleared supernatants were aliquoted and stored at -80°C. Protein concentration was determined by the BCA Assay Kit (Pierce II, USA).

Experimental Design of Western Blot Analysis and quantification

Samples were run on 10% SDS-polyacrylamide gels and transferred to nitrocellulose membrane at 400mA for 2h at 4°C. Membranes were blocked for 1h in 5% milk in TBST (TBS + 0.1% Tween-20). Primary antibodies used were: pS473Akt (dilution 1:5000; CST4060 Cell Signaling Technology), pan-Akt (dilution 1:5000; CST9272 Cell Signaling Technology), pSer9GSK3β (dilution 1:5000; CST9323 Cell Signaling Technology), pS6 (dilution 1:1000; CST2211, Cell Signaling Technology), S6 (dilution 1:10000; CST2217, Cell Signalling Technology), GAPDH (dilution 1:10000; CB1001, Sigma-Aldrich), α-tubulin (dilution 1:5000, T6199, Sigma-Aldrich). Secondary antibodies used were: goat Anti-Rabbit IgG antibody (H+L), Peroxidase (dilution 1:5000, PI-1000, Vector Laboratories), horse Anti-Mouse IgG antibody (H+L), Peroxidase (dilution 1:5000, PI-2000, Vector Laboratories). Membranes were incubated with primary antibodies diluted in 5% milk in TBST, overnight at 4°C with shaking. The membranes were rinsed 3 times for 10 min each time in TBST and then placed in the appropriate secondary antibody for 1h at room temperature with shaking. The membranes were rinsed again 3

times for 10 min each time, incubated with enhanced chemiluminescence substrate (Clarity, , USA) and the signal was detected and visualized with a ChemiDocTMXRS+ imaging system (BioRad, USA).

For comparisons between controls and FEP patients, samples were analyzed in four consecutive rounds with different control and patient samples run on the same gel together with external standard (ES) samples (two aliquots of 10 μg lysate from human A549 cells per gel). For the comparison of controls and patients (data in figures 1 and 2) we included samples from 36 patients and 36 controls. Some control and FEP patient samples were run and analyzed in more than one gel and in these cases we averaged all measurements for the final value. We routinely cut membranes after transfer in two parts with the lower MW part processed for pS6, S6 or GAPDH blots and the upper higher MW part used for pAkt, Akt, pGSK3 blots. In some experiments, we cut lower or upper parts of gels containing different groups of samples and transferred the gel strips onto the same membrane (see representative uncropped images of blots in Supplementary figure 1). To ensure reproducibility and comparisons between different rounds of analysis we included in every round samples from one or two preselected control subjects in every gel. For the comparison of patients at baseline and after APD treatment (figure 3) we used samples from 32 patients because four patients were not available for the follow up analysis. For this dataset, all samples were run in a strict paired fashion with baseline and APD treatment samples from the same patient run on the same gel together with external standard samples. We expressed each normalized phospho-specific signal as a ratio against total expression of GAPDH (for pAkt and pGSK3β) and S6 (for pS6), which were measured on the same gel (for pAkt/GAPDH and pGSK3β/GAPDH pairs) or in sister gels (for pS6/S6 pairs) and normalized accordingly to external standards. We also included measurements for α-tubulin on the pS6 and S6 gels in this dataset (figure 3). Membranes were routinely probed for pAkt or pGSK3 and subsequently for GAPDH, or S6 or pS6 and subsequently for α-tubulin (representative uncropped images are shown in Supplementary figure 1). The final dataset was not z-score transformed for the analysis in figure 3 and Supplementary Tables 3 and 4.

Multivariate exploratory analysis of phosphorylation values

The final dataset of pAkt, pGSK3β and pS6 phosphorylation values for 36 control and 36 FEP patient samples patients was imported into R (R version 4.1.0) using Rstudio (Version 1.4.1717) as continuous variables. After confirming, through frequency histograms and Shapiro-Wilk normality tests (p-value for all variables was <0.05), that none of the three variables follow a normal distribution, the variables were standardized as Z-scores and were used for all subsequent analyses.

Differences in phosphorylation levels of the three kinases between cases and controls were summarised into boxplots for each kinase, and non-parametric Mann–Whitney U tests were performed to infer statistical significances. Scatterplot matrices were plotted to investigate correlations. For each pair, a Spearman's test of association was applied to calculate the correlation estimate, while association p-values were calculated via the asymptotic approximation. Plots were generated using ggplot R package. Code, figures and detailed information on the analysis can be found on GitHub (https://github.com/digrigor/Schizo-FEP-Akt_paper_2021).

Regression analysis

A logistic regression using the logit distribution was calculated to assess the effects of pAkt, pGSK3 and pS6 phosphorylation values (continuous independent predictors) to the sample state (control vs FEP; binary dependent variable). The data was modelled using the "binomial" family of R's glm() function (phenotype ~ pAkt_zscore + pGSK3_zscore + pS6_zscore). The fitted model was tested to ensure it fulfils the assumptions of the logistic regression: 1) The outcome is a binary variable: FEP patient vs control. 2) There is a linear relationship between the logit of the outcome and each predictor variables: All variables are quite linearly associated with the outcome in logit scale. 3) There are no influential values in the continuous predictors: There are no standardized residuals >= 3 in our models, our data therefore do not have influential data points. 4) There are no high intercorrelations (multicollinearity) among the predictors for the fitted model: VIF (Variable Inflation Factors) was calculated for all the predictors and no values greater than 5 were observed showing no multicollinearity in the data. The logit regression equation is:

$$\log\left(\frac{p}{1-p}\right) = -0.19 + (-1.5) * pGSK3z + 1.21 * pS6z + 0.73 * pAktz$$

Where p is the is the predicted probability of the phenotype to be FEP, while $pGSK3z$, $pS6z$ and $pAktz$ are the z-scores of the GSK3, S6 and Akt phosphorylation values, respectively. The regression analysis reveals that all three variables significantly affect the state of the sample (control or FEP) (Supplementary Table 1), further validating the high pGSK3β/low pS6 phosphorylation pattern in FEP patients, with slightly lower pAkt values.

To investigate whether the interaction between the three continuous predictors also have an effect we fit a logistic regression model including the same pAkt, pGSK3 and pS6 variables as well as all the interactions between them using the "binomial" family of R's glm() function (phenotype ~ pAkt_zscore * pGSK3_zscore * pS6_zscore). The analysis revealed that the interaction between the variables do not have any effect (Supplementary Table 2).

Assessment of reproducibility in western blot analyses

Reproducibility was assessed by running the same samples of external standard and preselected control subjects in different gels and in different rounds. For the external standard, the calculated relative coefficient of variation was 7.5% for pAkt (n=10 from 5 different gels) and 7.8 % for pGSK3 (n=16 from 8 different gels) from a representative round. Accuracy of the densitometric analyses was assessed by performing dual measurements of ECL signals by two different researchers. The calculated relative coefficient of variation was 8% for the baseline vs after treatment pAkt and pGSK3 datasets. External standard sample was validated by running different amounts of protein (2, 5 and 10µg) on the same gel and calculating the final normalized pAkt. pGSK3β and pS6 values. The amount of protein did not substantially change the final values which were pAkt = 0.74±0.06 (n=6), pGSK3β =1.65±0.17 (n=6), and pS6=0.71±0.09 (n=6) for a representative experiment.
